# Acupuncture at GV20 and ST36 Improves the Recovery of Behavioral Activity in Rats Subjected to Cerebral Ischemia/Reperfusion Injury

**DOI:** 10.3389/fnbeh.2022.909512

**Published:** 2022-06-14

**Authors:** Yang Yang, Peiying Deng, Yingkui Si, Hong Xu, Jianmin Zhang, Hua Sun

**Affiliations:** ^1^Department of Traditional Chinese Medicine, Peking Union Medical College Hospital, Chinese Academy of Medical Sciences, Peking Union Medical College, Beijing, China; ^2^CAMS Key Laboratory for T Cell and Immunotherapy, State Key Laboratory of Medical Molecular Biology, Department of Immunology, School of Basic Medicine, Institute of Basic Medical Sciences, Chinese Academy of Medical Sciences, Peking Union Medical College, Beijing, China

**Keywords:** acupuncture, ischemic stroke, electroacupuncture, behavioral activity, neurovascular regeneration

## Abstract

Traditional acupuncture and electroacupuncture (EA) have been widely performed to treat ischemic stroke. To provide experimental support for the clinical application of acupuncture to ameliorate post-stroke sequelae, in this study, we investigated the therapeutic effect of acupuncture and EA on CIRI following middle cerebral artery occlusion (MCAO) in rats. The animals were randomly divided into five groups: sham-operated (S), model (M), traditional acupuncture (A) treatment, electroacupuncture (EA) treatment, and drug (D; edaravone) therapies. Neurological behavioral characteristics (neurological deficit score, forelimb muscle strength, sensorimotor function, body symmetry, sucrose consumption, and mood) were examined in all the groups on days 1, 3, 5, and 7 after reperfusion. Expressions of vascular endothelial growth factor (VEGF) and hypoxia-inducible factor-1α (HIF-1α) were detected by immunohistochemistry. Both acupuncture and EA significantly reduced neurological deficits and improved forelimb muscle strength, sensorimotor function, body symmetry recovery, and neurovascular regeneration in the rats after ischemia/reperfusion injury. The efficacies of both acupuncture and EA were comparable to that of edaravone, a commonly used medicine for stroke in the clinic. Thus, our data suggest that acupuncture and EA therapy at acupoints GV20 and ST36 might represent alternative or complementary treatments to the conventional management of ischemic stroke, providing additional support for the experimental evidence for acupuncture therapy in clinical settings. In summary, EA might provide alternative or complementary treatment strategies for treating patients with apoplexy in the clinic. However, potential mechanisms underlying the role of acupuncture require further investigation.

## Introduction

Ischemic cerebrovascular disease has high morbidity and mortality rates worldwide and accounts for more than 80% of all stroke cases, with a multifactorial pathology characterized by different events evolving over time (Wu et al., 2019; Zhou et al., 2019; Webb and Werring, 2022). Reperfusion damage occurs during bloodstream recanalization after a period of cerebral ischemia, continuing even after blood flow is restored. Previous studies on motor and sensory disorders after brain ischemia have revealed the neuroprotective effect of acupuncture on rats with cerebral ischemia/reperfusion injury (CIRI) (Cui et al., 2020; Kang et al., 2021), but only a few studies have examined the effect of acupuncture on neurobehavioral function and neurovascular regeneration. In clinical settings, acupuncture has significantly ameliorated physical dysfunction in patients with stroke sequelae (Chang et al., 2019; Du Y. et al., 2020). Behavioral testing paradigms have been used to evaluate the effect of acupuncture on post-stroke sequelae.

Acupuncture is widely performed to treat various neurological diseases because of its simple operation, high safety, and few side effects. Studies have also provided evidence that acupuncture potentially promotes stroke rehabilitation (Shao et al., 2020; Yao et al., 2020). Electroacupuncture (EA) is a complementary combination therapy consisting of traditional acupuncture and electrical stimulation. EA treatment effectively attenuates inflammatory injury and exerts a neuroprotective effect on ischemic stroke (Deng et al., 2022). We reported that EA treatment at the “Baihui” (GV20) and “Zusanli” (ST36) acupoints alleviated neuronal injury and reduced the infarct volume in rats with CIRI (Wang et al., 2019; Chen et al., 2020; Zhang Y. M. et al., 2021). Both acupuncture and EA are promising clinical therapies for ischemic stroke, although larger and more rigorous studies are needed.

Here, we performed a series of comprehensive behavioral tests to assess the efficacy of acupuncture and EA with GV20 (Baihui) and ST36 (Zusanli) in promoting the recovery of behavioral function in a classical ischemic stroke model of middle cerebral artery occlusion (MCAO). After first confirming that our animal model of CIRI induced by MCAO was successfully established, we evaluated the effects of acupuncture and EA on these rats by determining their mNSSs, examining limb sensorimotor ability, movement coordination, integration ability, and mood using grip strength, corner, cylinder, sucrose preference tests, and testing vascular endothelial growth factor (VEGF) and hypoxia inducible factor-1α (HIF-1α) proteins by immunohistochemistry. EA and acupuncture do improve sensorimotor ability, movement coordination, integration function, and neurovascular regeneration.

## Materials and Methods

### Animals

Healthy adult (8 weeks old) male Sprague-Dawley rats (*n* = 65) weighing 220–250 g were housed in an environmentally controlled room (Lee et al., 2014; Seunghoon et al., 2014; Hu et al., 2018). The temperature was maintained at 22 ± 2°C, and a 12-h light/dark cycle was used. Food and water were provided *ad libitum*. All procedures were performed in accordance with the guidelines of the Ethics Committees of Peking Union Medical College Hospital (PUMCH; Beijing, China) and the Chinese Academy of Medical Sciences (Beijing, China) as well as the Guide for the Care and Use of Laboratory Animals (National Institutes of Health, Bethesda, MD, United States). In addition, the Ethics Committees of PUMCH and the Chinese Academy of Medical Sciences specifically approved this study (permit no. D-002). All efforts were made to minimize animal suffering and the number of animals employed.

### Rat Model of Cerebral Ischemia/Reperfusion Injury

We tested the cerebral blood flow in the rat model of CIRI induced by MCAO by laser Doppler blood flowmetry to ensure a homogeneous and stable model. Focal cerebral ischemia was induced as described previously but with slight modifications (Longa et al., 1989). Briefly, anesthesia was induced in the rats in an anesthesia induction box filled with a mixture of oxygen and 5% isoflurane at a flow rate of 25 ml/min. The level of isoflurane was decreased to 2.5% at a flow rate of 5 ml/min for maintenance of a stable level of anesthesia. Body temperature was monitored and maintained at 37°C using a reactive heating pad. After shaving and disinfecting the surgical site by applying alternating solutions of Betadine and ethanol, a small vertical cut (approximately 1 cm) was made along the midline of the calvarium. Muscles attached to the temporal bone were excised approximately 2 mm posterior to the anterior fontanelle and 6 mm laterally to maintain a clean surgical field. The skull was opened with a cranial drill for the placement of the optical fiber to allow for laser Doppler flowmetry to be conducted and detect cerebral blood flow in the ischemic area. After regional cerebral blood flow became stable, MCAO was performed. The rats were placed in the supine position. The neck was incised along the midline for approximately 1.5 cm, and the right common carotid artery, the internal carotid artery, and the external carotid artery were exposed. A suture material (3400AAA; Guangzhou Jialing Biotechnology Co., Ltd.) was slowly advanced into the internal carotid artery through the external carotid artery stump to approximately 18–20 mm beyond the carotid artery bifurcation until the origin of the middle carotid artery was ligated. The cerebral blood flow value decreased rapidly to less than 70% of the baseline value. The incision was covered with a saline-soaked gauze containing gentamicin, and cerebral blood flow values were recorded. The ligature was removed 90 min later to allow for reperfusion, and cerebral blood flow was continuously recorded until the value was stable.

### Experimental Groups and Treatments

All the animals were randomly divided into five groups: sham (S), model (M), acupuncture (A), electroacupuncture (EA), and drug (D; edaravone) (*n* = 13 per group). The latter four groups underwent MCAO, with a decrease of greater than 70% in cerebral blood flow rates and a blood flow recovery of 50% after reperfusion. Groups A, EA, and D were treated with different therapies one time daily. The rats in group S were subjected to the same surgical procedures but without suture insertion into the internal carotid artery. The behavioral activities of all the rats were assessed on days 1, 3, 5, and 7 after reperfusion.

The rats in groups A and EA received acupuncture treatments by needling with disposable sterile acupuncture needles (diameter 0.32 mm; length 25 mm; Tianjin Huahong Medical Company, Tianjin, China) at acupoints GV20 (Baihui) and left ST36 (Zusanli) in a 20-min session once daily. GV20 is located on the top of the head at the intersection of the midsagittal line with the line connecting the two ear apexes. ST36 is located 5 mm distal to the head of the fibula beneath the stifle and 2 mm lateral to the tibial tuberosity. Two electrodes were attached to the head of the rats in the EA treatment group for acupuncture and continuous-wave stimulation at a frequency of 2 Hz (intensity 1 mA) for 20 min using an electroacupuncture device (KWD-808 II; Great Wall Brand, Baoding, China). After CIRI, the rats in group D were administered an intraperitoneal injection of edaravone (0.3 mg/kg; Nanjing Simcere Dongyuan Pharmaceutical Co., Ltd., Nanjing, China) one time daily.

### Evaluation of Neurological Damage

The mNSSs of rats in the five groups were determined (Andrews et al., 2019), and the motor, sensory, and reflex scores were recorded at various times following reperfusion to assess the abilities of the three treatments to ameliorate the neurological damage induced by CIRI.

### Grip Strength Test

The rats in each group were placed over a metal mesh grid connected to a force transducer. The rats gripped the grid with their forelimbs and were tugged gently until they released the grid. The grip strength of both forelimbs was measured three times, and the average was recorded as the final value (Glab et al., 2022).

### Corner Test

The apparatus for the corner test consists of two vertical boards placed at a 30° angle, with a narrow gap between the two plates to attract the rats into this “corner” (Lu et al., 2021). A rat was placed in the apparatus and walked into the corner. The subsequent rearing or turning of the rat toward either side was recorded.

Rats with CIRI preferentially turned away from the corner by leading themselves with the non-impaired side of the body. Each rat was evaluated ten times with a 1-min interval between tests. However, if a rat did not raise its forelimbs, the test was repeated. The laterality index was calculated as the number of right turns-the number of left turns/total number of turns.

### Cylinder Test

The cylinder test was conducted to evaluate the motor function and asymmetry of forelimb usage in post-ischemic rats (Asgari Taei et al., 2021). The rats were placed in a Plexiglas cylinder (diameter 20 cm, height 30 cm) on a clean desktop, and forelimb use was observed for 5 min. When the rats stood fully upright (during vertical exploration) and moved laterally, touching the cylindrical wall with their forelimbs to maintain their center of gravity, the number of times the animals used their left or right or both forelimbs simultaneously was recorded. For calculating asymmetric limb use score, “I” represents the number of times the right forelimb was used, “C” the number of times the left forelimb was used, and “B” the number of times both forelimbs were used simultaneously. The asymmetric limb use score = {I/(I + C + B)}-{C/(I + C + B)}. The normal rats turned toward the left side 50% of the time and toward the right side 50% of the time. Thus, the more severe the MCAO-induced injury, the higher the asymmetric limb use score. Behavior was assessed at regular intervals by an observer who was blinded to the treatment status.

### Sucrose Preference Test

We trained the rats to drink sucrose water in a quiet room. Each rat was placed in a cage with two bottles of water. They were initially trained for 48 h. For the first 24 h, both bottles contained 1% sucrose water and were provided to the rats. During the next 24 h, one bottle contained 1% sucrose water, and the other bottle was filled with tap water. Then, we started to formally test the rats. After 23 h of fasting, one bottle of 1% sucrose water and one bottle of tap water were offered. The two bottles were weighed 60 min later, and the amount of liquid consumed by the rats was recorded. The sucrose preference score was calculated as sucrose consumption/total liquid consumption × 100 (Liu et al., 2018; Yin et al., 2021).

### 2,3,5-Triphenyltetrazolium Chloride Staining

The 2,3,5-triphenyltetrazolium chloride (TTC) staining method was used to determine the infarct volume in the ischemic brains on day 3. The white areas in tissue slices stained with TTC indicate ischemia. In the present study, the MCA was occluded on the right side of the rats. Thus, brain regions supplied by the MCA, especially the cortex and the striatum in the right hemisphere, were white, and the rats showed left-side paralysis.

### Immunohistochemistry Analysis

On day 7, the paraffin-embedded brain tissue was cut into 6-μm-thick sections, deparaffinized with xylene, and dehydrated with gradient ethanol. A sodium citrate buffer (pH 6) was used for antigen retrieval. Endogenous peroxidase activity was blocked with 2% hydrogen. After blocking the activity with 5% goat serum for 30 min at room temperature, the sections were incubated with antibodies (1:1,000) overnight at 4°C. Then, the sections were incubated with HRP-conjugated secondary antibodies for 60 min at room temperature. Finally, visualization was performed using the DAB staining solution.

### Statistical Analysis

The GraphPad Prism 8 software (GraphPad Software, San Diego, CA, United States) was used. Data are presented as the means ± SD. Statistical analyses were performed by Student’s *t*-test, one-way analysis of variance (ANOVA), or repeated-measures two-way ANOVA with Tukey’s *post hoc* test for group comparisons. Levels of statistical significance were indicated with asterisks. The *p-*values < 0.05 were considered statistically significant.

## Results

### Establishment of Middle Cerebral Artery Occlusion-Induced Cerebral Ischemia/Reperfusion Injury

**FIGURE 1:**
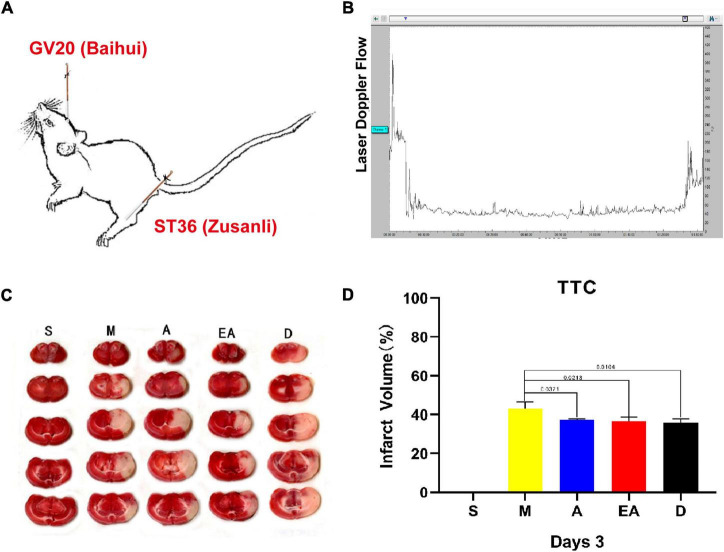
Establishment of the rat middle cerebral artery occlusion (MCAO) model. **(A)** Detailed acupoint locations used for acupuncture and electroacupuncture. **(B)** Laser Doppler flux measured over the lateral parietal cortex in the core of the ischemic region of MCAO rats. **(C)** Representative images of 2,3,5-triphenyltetrazolium chloride (TTC) staining in rat brain slices from all groups (*n* = 3). **(D)** Quantification of infarct volumes in the whole hemisphere after 90 min of MCAO in rats. Data are presented as the mean percentage of the entire ischemic hemisphere ± SD.

The rats were subjected to transient occlusion of the middle cerebral artery (MCAO) for 90 min to establish a rat model of cerebral ischemia/reperfusion injury. Over the 90-min period of occlusion, cortical perfusion was monitored by laser Doppler flowmetry. Detailed acupoint locations are shown in Figure 1A. As shown in Figure 1B, a stable and significant reduction in cortical perfusion (over 70% of the baseline) is observed throughout the occlusion period and is recovered to approximately pre-ischemic levels immediately upon the removal of filaments. TTC staining of the brain sections was conducted to assess the ischemic lesions in the cerebral cortex and the striatum in the right hemisphere of the rats and to further validate the model of MCAO on day 3. In the MCAO group, ischemia-induced infarct volumes were significantly larger at 72 h after stroke than those in the sham group (Figure 1C). However, treatment with A, EA, and edaravone markedly decreased the ischemia-induced infarct volume, and no significant differences were observed among the three treatment groups (Figure 1D). These results suggest that MCAO-induced cerebral ischemia/reperfusion injury was successfully established.

### Neurological Function

**FIGURE 2:**
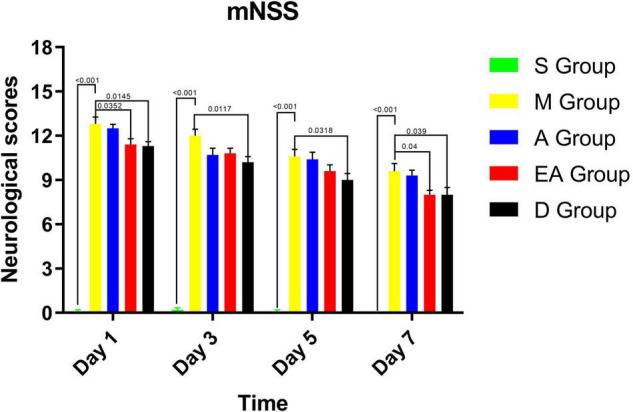
Modified neurological severity scores (mNSSs) in MCAO rats. Values represent means ± SD. *n* = 10.

The rats were subjected to MCAO for 90 min and then received acupuncture or electroacupuncture treatment to investigate the neuroprotective effect of acupuncture at GV20 and ST36 on rats with cerebral ischemia/reperfusion injury. Neurological deficits were assessed using the mNSS on days 1, 3, 5, and 7 after reperfusion. The mNSS is a composite of sensory, motor, reflex, and balance test scores and is graded on a scale of 0–18 points, as previously described (Chen et al., 1996; Andrews et al., 2019). A higher mNSS indicates more severe neural damage: the normal score is 0, and the maximal deficit score is 18. In this study, the mNSS was classified into three levels: severe (13–18 points), moderate (7–12 points), and mild (less than 6 points) deficits. No loss of neurological function was observed in the sham rats (Figure 2). The rats in group M showed the highest mNSSs daily among the other groups. Group EA significantly improved the neurological function compared with group M (Figure 2). Although electroacupuncture resulted in lower scores than acupuncture treatment, the difference was not statistically significant. Based on these results, electroacupuncture exerts a similar therapeutic effect on behavioral recovery after ischemia/reperfusion injury. Intraperitoneal injection of edaravone one time daily exerted the best therapeutic effect on behavioral recovery (Figure 2).

### Muscle Strength

**FIGURE 3:**
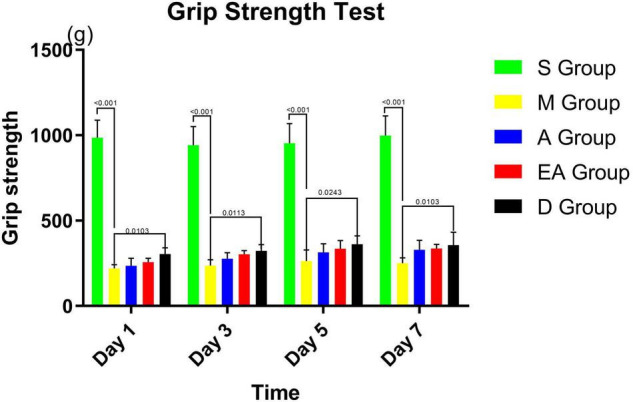
Grip strength scores of MCAO rats. Values represent means ± SD. *n* = 10.

We performed grip strength tests to assess the therapeutic effect of acupuncture treatment on the recovery of muscle strength after ischemia/reperfusion injury. The muscle strength scores were significantly reduced in the MCAO rats compared with the sham rats (Figure 3). However, the muscle strength scores were significantly recovered in groups EA, A, and D on days 3, 5, and 7 after treatments compared with group M without any treatment. No significant difference was observed in grip strength among the rats in groups EA, A, and D (Figure 3).

### Sensorimotor Function and Motion Integration Function

**FIGURE 4:**
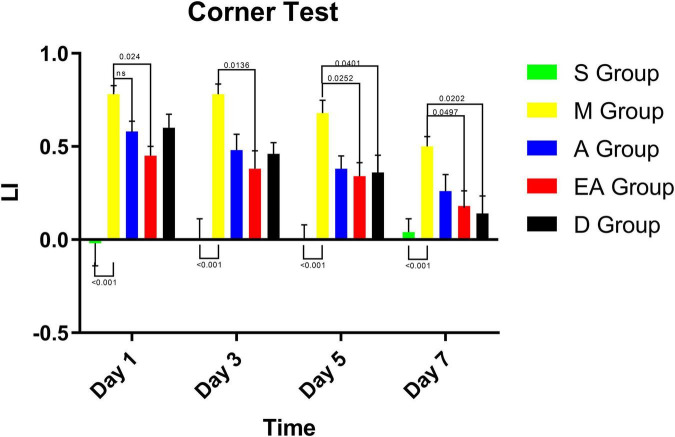
Corner test results for MCAO rats. Values represent means ± SD. *n* = 10.

We also performed corner tests to assess the therapeutic effects of acupuncture on the recovery of sensorimotor function and motion integration function. We found that the laterality index in group S was approximately zero (Figure 4), indicating normal sensorimotor function and postural symmetry in the sham rats. However, group M showed the highest laterality index score (Figure 4), suggesting significant deficits in sensorimotor function and postural symmetry after MCAO. Either acupuncture, electroacupuncture, or intraperitoneal injection of edaravone significantly reduced the laterality index on days 1, 3, 5, and 7 (Figure 4), indicating a therapeutic effect of the treatments on the recovery of sensorimotor function and motion integration function.

### Asymmetric Limb Use

**FIGURE 5:**
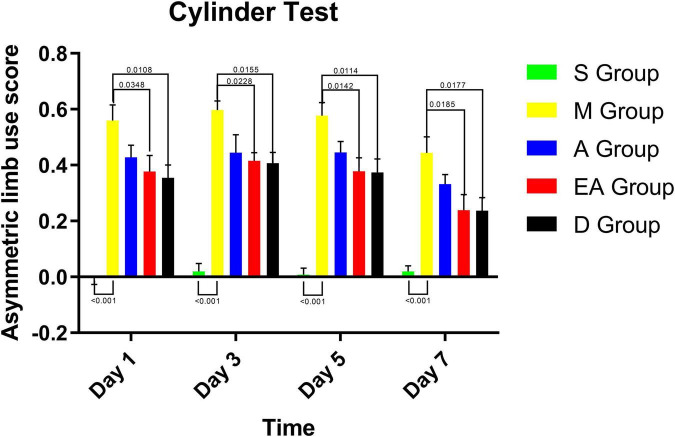
Cylinder test results for MCAO rats. Values represent means ± SD. *n* = 10.

The cylinder test was conducted to evaluate spontaneous forelimb use. Forelimb use was symmetrical in group S, as shown by an asymmetric limb use score of approximately zero, whereas a significant asymmetric limb use was observed in group M (Figure 5). After receiving treatments, the rats in groups A, EA, and D showed a decreasing trend in the asymmetric use of their forelimbs. The use of the affected limb by the rats in groups EA and D was significantly recovered compared with that of the rats in group M on all the days tested. However, the cylinder test did not reveal significant differences among the A, EA, and D groups.

### Depression-Like Behavior

**FIGURE 6:**
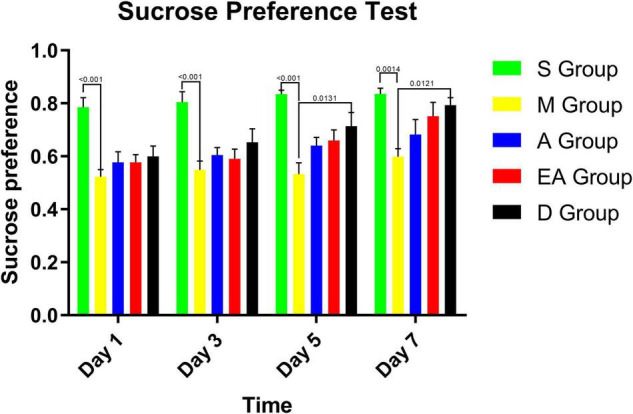
Sucrose preference test results for MCAO rats. Values represent means ± SD. *n* = 10.

In addition to motor and sensory disorders after brain ischemia, various degrees of depression occurred. The sucrose preference test was conducted to evaluate a depression-like behavior in the rats after CIRI. As shown by the results of the sucrose preference test (Figure 6), the rats in group M consumed significantly less sucrose water than those in group S. After receiving treatments, the rats in group D had significantly higher sucrose preference scores than the untreated group M on days 5 and 7. Although the rats in groups A and EA tended to increase their sucrose consumption over time, the difference was not statistically significant.

### Neurovascular Regeneration Capacity

**FIGURE 7:**
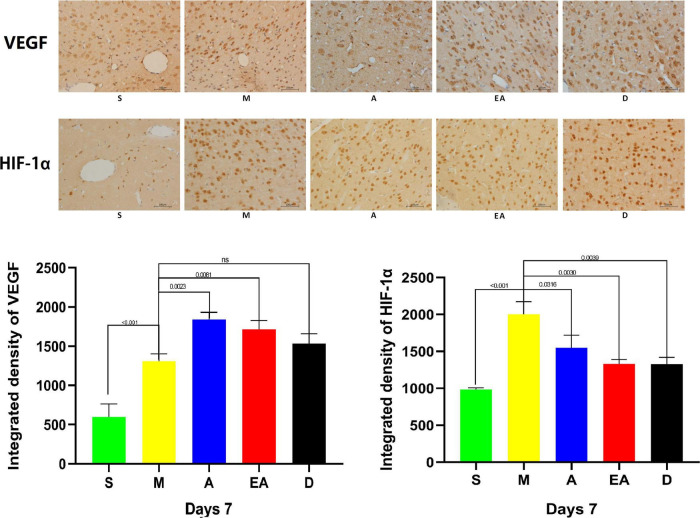
Expression of vascular endothelial growth factor (VEGF) and hypoxia-inducible factor-1α (HIF-1α) in the brain tissue of MCAO rats on day 7 after cerebral ischemia/reperfusion injury (CIRI). Scale bars = 100 μm. Values represent means ± SD. *n* = 5.

Recent studies demonstrated that the degree of increased vascular density in brain tissues after stroke is closely related to the prognosis of patients with stroke and that angiogenesis after CIRI is tightly regulated by VEGF (Du J. et al., 2020; Wang L. et al., 2021). HIF-1α is an important transcription factor that maintains tissue homeostasis and promotes angiogenesis after stroke (Yu et al., 2021; Zhang C. et al., 2021). Therefore, we detected the expression of VEGF and HIF-1α in the brain of the rats on day 7 after treatment. The results of immunohistochemistry showed that the levels of VEGF and HIF-1α were increased in group M compared with group S. After treatment with acupuncture, electroacupuncture, or intraperitoneal injection of edaravone, the expressions of VEGF were increased, whereas the expressions of HIF-1αwere significantly decreased. No significant difference was observed between these treatments (Figure 7).

## Discussion

Traditional Chinese medicine theory suggests that ischemic cerebrovascular disease belongs to the “stroke” category and that its main pathogeneses are virtual product loss, *yin* and *yang* imbalance, and blood against chaos, which ultimately cause blockage and cerebral ischemia (Miao et al., 2017; Wu et al., 2017; Li et al., 2019). Acupuncture can clear blockages in the brain vascular system, regulate *yin* and *yang* imbalance, and improve vital *qi* in the body (Lou et al., 2020; Zhao et al., 2020; Wang C. C. et al., 2022). In the present study, we chose acupoints GV20 and ST36 based on the theory of meridians and acupuncture points. GV20 belongs to the governor vessel, which may connect all *yang* vessels in the body and functions to regulate local *qi* and blood, and modulates the balance between *yin* and *yang* (Zhang et al., 2014). After stimulation, GV20 returns the body to normal functions, dispersing local *yang* (Xu et al., 2014). ST36 belongs to the stomach meridian, which is rich in both *qi* and blood. Thus, it is considered an acupuncture point that plays a role in the recovery of paralysis (Cui et al., 2021; Deng et al., 2022). The combined use of these two acupuncture points is effective at dredging channels and collaterals, modifying the blood and *qi*, and balancing *yin* and *yang*.

Electroacupuncture therapy was developed from traditional acupuncture, adding electrical stimulation to acupuncture therapy. Many studies have focused on mechanisms underlying the neuroprotective effect of electroacupuncture therapy, and some have shown that electroacupuncture improves neurological behavioral outcomes (Deng et al., 2022), increases cerebral blood flow in the ischemic area (Mei et al., 2020), inhibits neural inflammation and neural hypoxia (Xu et al., 2018; Long et al., 2019), and promotes angiogenesis and neurogenesis (Shi et al., 2017; Chen et al., 2020; Li et al., 2020, 2021). Therefore, we selected GV20 and the left ST36 to treat the hemiplegia caused by CIRI in the rats. Electroacupuncture and acupuncture manipulations provide two different types of stimulation. Electroacupuncture depends on an electric current to stimulate acupuncture points, whereas the effect of acupuncture is produced by mechanically inserting and lifting a needle, even by twisting, to stimulate acupuncture points (Wang et al., 2020; Wang S. J. et al., 2022). The advantages of electroacupuncture over acupuncture are that it more accurately sets stimulation parameters and is less labor-intensive than acupuncture. Edaravone is a neuroprotective agent. It eliminates free radicals and inhibits lipid peroxidation, thereby inhibiting oxidative damage to brain cells, endothelial cells, and nerve cells and reducing cerebral edema and brain tissue damage (Li and Liu, 2021; Xie et al., 2021; Yang et al., 2021). Therefore, we used this medicine as a positive control.

The rats with MCAO-induced CIRI in the present study showed the following symptoms: limb paralysis, decreased muscle strength, affected limb hypoesthesia, decreased utilization of the affected limb, decreased ability to integrate body movement, and other nerve dysfunction-related behaviors. The mNSS provides a more detailed neurological assessment than those developed by Bederson and Longa (Shi et al., 2020). The mNSS includes scores for motor and sensory function and balance and scores reflecting the deep sensation and superficial reflexes of nerves. It is a comprehensive rating scale that is an indispensable indicator for evaluating the success of CIRI modeling and treatments (Wen et al., 2017; Andrews et al., 2019).

In the present study, the extent of damage to the forebrain and the striatum was detected in the corner test. The laterality index has been used to directly observe the therapeutic effects on the body, validating the repair of the damage in the forebrain and the striatum of rats treated with acupuncture. The cylinder test has been conducted to determine asymmetry in forelimb use, forelimb strength, motor integration, and behavioral improvement after cerebral ischemia. The sucrose preference test has been conducted to examine depression-like and anxiety-like behaviors in CIRI rats (Wang A. R. et al., 2021). Based on our results, the rats displayed behaviors characteristic of depression and anxiety after CIRI, although acupuncture therapy did not significantly alter these behaviors in the short term. Neurovascular regeneration condition was examined by detecting the expression of VEGF and HIF-1α. The results of our experiments indicated positive effects of acupuncture, electroacupuncture, and edaravone treatments on the neurological deficit score, forelimb muscle strength, sensorimotor function, body symmetry, and expression of VEGF and HIF-1α in rats with CIRI. Acupuncture and electroacupuncture therapies significantly improved limb sensorimotor function, movement integration, and neurovascular regeneration.

In summary, our results indicate that acupuncture and electroacupuncture therapies at GV20 and ST36 might represent alternative or complementary treatments to the conventional management of ischemic stroke and provide further support for the experimental evidence of acupuncture use in clinical settings.

## Data Availability Statement

The original contributions presented in this study are included in the article/supplementary material, further inquiries can be directed to the corresponding author/s.

## Ethics Statement

The animal study was reviewed and approved by the Ethics Committees of Peking Union Medical College Hospital (PUMCH; Beijing, China) and Chinese Academy of Medical Sciences (Beijing, China) as well as the Guide for the Care and Use of Laboratory Animals (National Institutes of Health, Bethesda, MD, United States).

## Author Contributions

YY, JZ, and HS designed and carried out the conception of the study. YY and YS performed the experiments. YY and PD contributed to the data analysis and drafting of the manuscript. PD, HX, and HS contributed to the data analysis and revision of the manuscript. YY and JZ contributed to the reagents, materials, and analysis tools. All authors contributed to the article and approved the submitted version.

## Conflict of Interest

The authors declare that the research was conducted in the absence of any commercial or financial relationships that could be construed as a potential conflict of interest.

## Publisher’s Note

All claims expressed in this article are solely those of the authors and do not necessarily represent those of their affiliated organizations, or those of the publisher, the editors and the reviewers. Any product that may be evaluated in this article, or claim that may be made by its manufacturer, is not guaranteed or endorsed by the publisher.
